# Physical therapy treatments incorporating equine movement: a pilot study exploring interactions between children with cerebral palsy and the horse

**DOI:** 10.1186/s12984-021-00929-w

**Published:** 2021-09-06

**Authors:** Priscilla Lightsey, Yonghee Lee, Nancy Krenek, Pilwon Hur

**Affiliations:** 1 Ride On Center for Kids (ROCK), Rockride Ln, Georgetown, TX 78626 USA; 2 Department of Mechanical Engineering, Texas A&M University, TAMU, College Station, TX 77843 USA; 3 School of Mechanical Engineering, Gwangju Institute of Science and Technology, Cheomdangwagi-ro, Gwangju, 61005 South Korea

**Keywords:** Hippotherapy, Equine assisted therapy, Interaction children with cerebral palsy, Functional mobility

## Abstract

**Background:**

Physical therapy treatments incorporating equine movement are recognized as an effective tool to treat functional mobility and balance in children with cerebral palsy (CP). To date, only a few studies examined kinematic outputs of the horses and children when mounted. In this pilot study, to better understand the effectiveness of this type of treatment, we examined the interaction between the horses and children with CP during physical therapy sessions where equine movement was utilized.

**Methods:**

Four children with CP participated in eight physical therapy sessions incorporating hippotherapy as a treatment intervention. Functional mobility was assessed using the Timed Up Go or the 10 m Walk Test. Inertial measurement unit sensors, attached to children and horses, recorded movements and tracked acceleration, angular velocity, and body orientation. Correlation between vertical accelerations of children and horses were analyzed. In addition, peak frequencies of vertical accelerations of children and horses were compared.

**Results:**

Functional tests modestly improved over time. The children’s movements, (quantified in frequency and temporal domains) increasingly synchronized to the vertical movement of the horse’s walk, demonstrated by reduced frequency errors and increased correlation.

**Conclusions:**

The findings suggest that as the sessions progressed, the participants appeared to become more familiar with the horse’s movement. Since the horse’s gait at a walk mimics the human gait this type of treatment may provide individuals with CP, who have abnormal gait patterns, an opportunity for their neuromuscular system to experience a typical gait pattern. The horse’s movement at the walk are consistent, cyclical, rhythmical, reciprocal and multi-dimensional, all of which can facilitate motor learning. The increased synchronization between horse and the mounted participant suggests that physical therapy utilizing equine movement is a viable treatment tool to enhance functional mobility. This study may provide a useful baseline for future work.

*Trial registration*Texas A&M University Institutional Review Board. IRB2018-0064. Registered 8 March 2018. Link: https://rcb.tamu.edu/humans/irb and https://github.com/pilwonhur/HPOT

## Background

The primary goal of any physical therapy treatment is to improve a patient’s functional ability [1]. Functional mobility is defined as the way a person moves within their environment on a daily basis to interact with society and family [2]. Healthcare providers frequently treat individuals with cerebral palsy who have deficits in functional mobility as well as in other domains. The diagnosis of cerebral palsy (CP) refers to a non-progressive lesion in the developing brain which affects a person’s ability to move [3]. CP is the most common cause of motor disability in children [2, 4, 5] and Kirby et al. [4] reported that the prevalence of CP is 3.3 per 1000 births in the United States, with 75–81% of those diagnosed with spastic CP. It often causes poor balance and muscle weakness [3]. These deficits lead to decreased postural control, which is essential for all movements [6, 7]. Further, poor balance adversely affects functional mobility which in turn affects activities of daily living [8]. Physical therapists work with this population to facilitate improved motor function to enhance daily life [9]. Therapy often spans years for individuals with CP, making it challenging for therapists to find a variety of effective, evidenced-based treatments that are also motivating for the patient over a long period of time. This study is intended to contribute an evidence-based treatment option for physical therapists, one that may be considered novel, enjoyable, and appealing when compared to traditional therapy techniques.

One treatment option that may benefit persons with CP is physical therapy incorporating equine movement, traditionally known as hippotherapy (HPOT) [10–16]. HPOT is a treatment strategy applied by licensed therapists or therapist assistants of physical, occupational, and speech therapy in which the equine movement is utilized and manipulated by the therapists to attain functional goals [10, 12, 14, 15, 17]. During HPOT, activities are based on the participant’s position and movement while mounted [15]. HPOT can be part of an integrated treatment plan that addresses functional limitations and impairments to facilitate functional skills [10, 12, 14]. Specific physical therapy goals for an HPOT session often include improving overall function, balance, and posture [10, 14]. Previous studies describe the benefits of HPOT and therapist-designed adaptive riding for children with CP, including improved gross motor function, dynamic balance, and trunk postural coordination [11, 12, 14–18]. In this study, the term HPOT will be used to refer to physical therapy sessions that incorporate equine movement as a therapy tool.

The principles of HPOT derive from the movements a horse provides to the individual astride the equine. Studies have been done to look at the kinematic movement patterns of the horse and rider. MacPhail et al. [13] used kinematic analysis to look at the pelvic movement of the horse and lateral trunk movements of riders; six with CP and seven with no disabilities. Kinematic analysis revealed that the horse’s pelvis appeared to move in a dual frequency sinusoidal curve pattern, as opposed to a simple sinusoidal curve, leading researchers to note that this more complicated movement pattern increased the need for postural adjustments of riders. The increased demand on the rider to respond to the movement imparted by the horse appeared to have facilitated typical equilibrium reactions in the two participants with CP. The researchers reported that normal equilibrium responses (using the children who were typically developing as the reference) were elicited in 65–75% of the responses for riders who had diplegic CP and 10–35% of the responses for riders with quadriplegic CP. The researchers concluded that for children with diplegic CP, it might be an effective way to elicit and practice sitting equilibrium reactions [13].

Haehl et al. [19] examined movement patterns using a camcorder to collect kinematic data on riders and horses. The investigators first looked at two children without special needs and tracked the kinematic relationship. They found that the riders demonstrated a biphasic movement pattern in reaction to the horse’s movements. Second, they examined two children with CP for 12 weekly HPOT sessions. Data found that the biphasic movement patterns seen in the typically developing children were approximated in the children with CP as the session progressed. Also, both participants with CP demonstrated enhanced coordination between upper and lower trunk, exhibiting the most overall postural stability during the final HPOT session. The researchers noted that the participants displayed “behavioral instability”—the chance to problem-solve, reorganize, and change postural coordination—a component to learning new movement strategies. Also, functional mobility improved in one child, whose transfers and ambulation skills were notably enhanced. The authors stated that novel, more efficient movement patterns may have arisen, replacing older, familiar patterns as a result of the opportunities for a child to explore new movement strategies during the HPOT session [19].

A study conducted by Garner and Rigby [20] quantitatively measured pelvic motion of six children without disabilities when riding a horse compared to walking on a stable, even surface. Five kinematic measures were taken, using motion capture systems to observe the inexperienced riders. The researchers focused on the pelvic motion of the participants, specifically: vertical, anterior-poterior, and lateral translations as well as pelvic twist and list angles. The participants rode each of the four horses at walk, then walked on foot, through the two observational spaces. Findings revealed that displacement amplitudes and up-and-down, forward-and-backward, and side-to-side translations were similar for both riding and walking [20]. Garner and Rigby concluded that, since a horse can impart movements similar to the human walking pattern to the pelvis of the rider, riding a horse may provide therapeutic benefits for persons with disabilities who cannot move in a typical gait pattern.

Goals for physical therapy treatments incorporating equine movement often relate to improving balance, posture, and overall function [10, 14]. Coordination and postural control are dynamic processes [19] which can be addressed during an HPOT session. This is significant since postural control is the ability to maintain equilibrium in the field of gravity [21]. Postural stability is also the basis for performing increasingly more difficult motor tasks [22]. The horse is a dynamic base of support and the repetitive movement during HPOT provides the rider with multiple opportunities to practice postural control and develop—then practice—new skills. Haehl, et al [19] and others [11–14, 17, 23] have noted that HPOT has positively influenced the functional mobility of children with movement disorders. The multidimensional movements of the equine that are imparted to the rider translates to improved gait and balance off the horse [23].

A study by Uchiyama et al. [24] used acceleration data to evaluate the similarity between the movements of children and horse based on the hypothesis that the horse’s pelvic movement during therapeutic riding sessions are similar to the human pelvic movement while walking. Three-dimensional accelerometers collected acceleration of both horses and humans walking for a three-minute period and stride-phase data was generated from foot movements. The results showed that the frequency peaks of human walking corresponded with those of the horse walking, especially during the stride-phase. The authors concluded that riding a horse at a walk provides sensory and motor input to the rider comparable to the human activity of walking, thus offering a potential treatment option for individuals with gait abnormalities [24].

While studies have shown potential benefits in enhancing functional mobility of the children with CP, it is still unclear how the enhancement is accomplished. Interaction between the children with CP and the horses is deemed to be the main enabler of the successful rehabilitation. However, these studies showing association between kinematics of horse movement and children’s movement with CP did not attempt to systematically examine how the interaction affects the functional mobility of the children with CP. The objectives of this study are to examine (1) how the use of HPOT in physical therapy treatments affects the functional mobility of the children with CP, (2) how physical therapy incorporating equine movement affects the interaction between the rider, i.e., children with CP, and the horse, and (3) how functional mobility correlates with the interaction.

## Methods

### Participants

This repeated-measure design study consisted of functional assessments and kinetic sensor measurements. A convenience sample of participants was recruited. Approvals of Institutional Review Board and Animal Use Protocol from Texas A&M University (TAMU) were obtained. Consent forms and signed releases were completed by parents of the participants. Inclusion criteria were:

- ages 2.5–14 years of age diagnosed with spastic cerebral palsy
- GMFCS (Gross Motor Function Classification System) level I, II, or III
- ability to reliably signal pain, fear, or discomfort and follow simple directions
- lack of or mild scoliosis
- no botulinum toxin treatments, orthopedic, or neurosurgery in the six months preceding initiation of HPOT sessions

Subjects were recruited from two Professional Association of Therapeutic Horsemanship International (PATH Intl.) Premier Accredited Centers: TAMU Courtney Cares in College Station, TX and ROCK in Georgetown, TX. Clients who were eligible for research participation according to the inclusion criteria were asked, under the guidance of their legal guardian, if they were interested participating.

In total, four subjects participated in the experiment. The first three subjects, all GMFCS Level II, had spastic hemiplegia CP. The fourth subject, GMFCS Level III, had spastic quadriplegia CP and used a rolling walker for assistance when ambulating (Table 1). GMFCS describes the gross motor function of persons with CP by using a five-level, simple grading system and is the most recognized and established functional classification measure for CP [25]. It was selected for the criteria as it provides a method of describing function that is quick, easy to use, and meaningful to health care professionals.

**Table 1:** Participant Demographics and Characteristics

Participant	Age (years)	Sex	GMFCS	Type of CP	Ambulation assistive device
1	2.5	F	II	Hemiparesis	None
2	4.3	M	II	Hemiparesis	None
3	12.5	F	II	Hemiparesis	None
4	10.8	F	III	Quadriparesis	Rolling walker

### Experimental protocols

#### Functional mobility tests

**Fig. 1:**
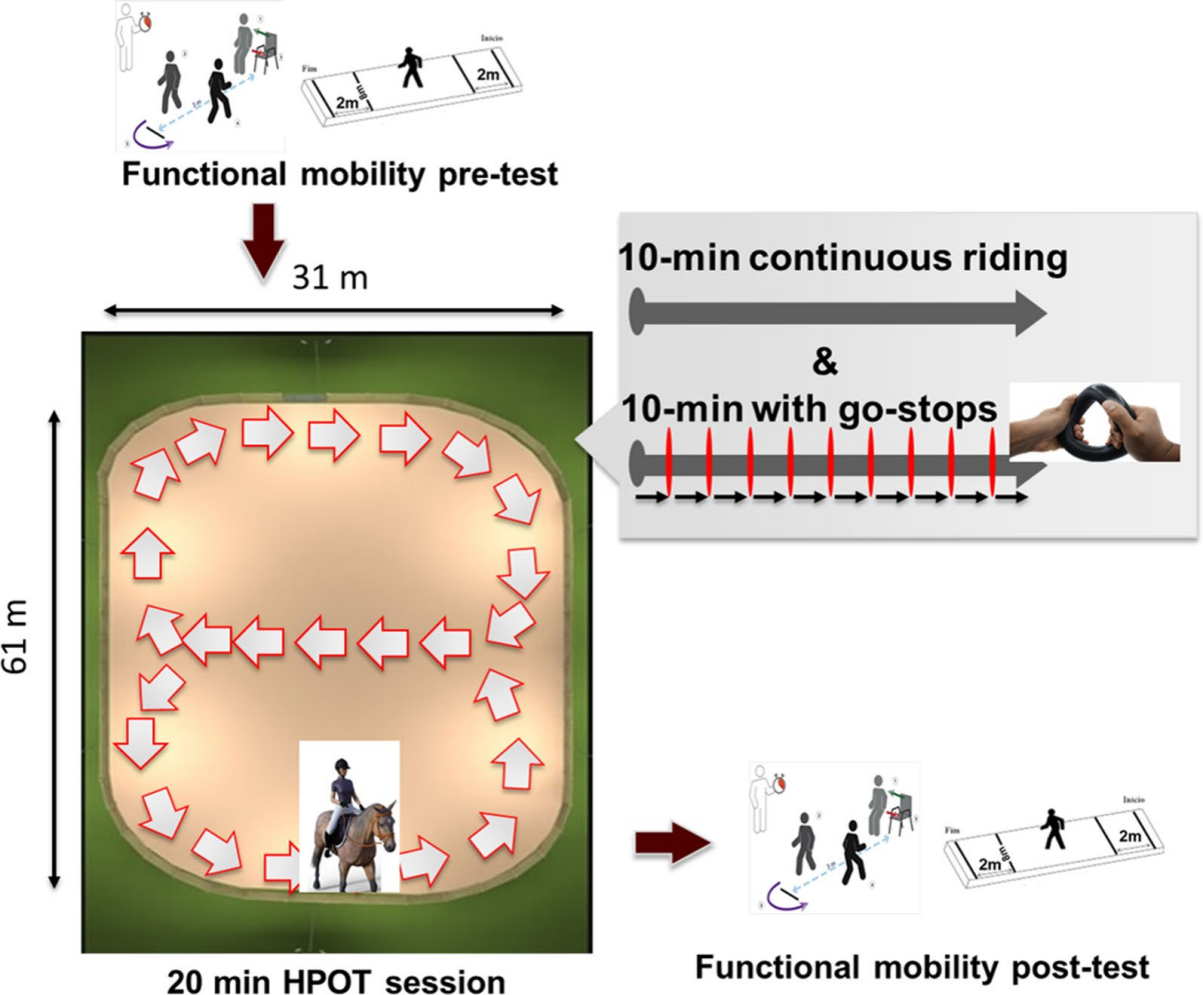
Experimental protocol. Functional mobility tests were performed before and after the HPOT sessions. Each 20-min HPOT session consisted of 10-min continuous riding and 10-min riding with multiple go-stops. The figure-of-eight patterns were made during the HPOT session

The experiment was conducted at two PATH International Premier Accredited Centers and at TAMU Parson’s Mounted Cavalry Headquarters. Data were collected on days one, four, and eight of the eight sessions, with functional assessments performed prior to and immediately after each HPOT session (Fig. 1). Tests that assess gait speed were chosen since it is a key indicator of performance in individuals with neurological disorders [26, 27]. The Timed Up and Go (*TUG*) measures the time it takes a child to stand up from a chair, walk 3 meters, turn around, walk back to the chair, and sit down. The *TUG* was used because it is commonly used measure to test dynamic and functional balance [28]. In children, the *TUG* is used to identify deficits in dynamic balance that may delay motor skill acquisition and could cause motor delay [28]. In addition, it has been shown to correlate well with other measures of balance, postural sway, and gait speed [29].

**Fig. 2:**
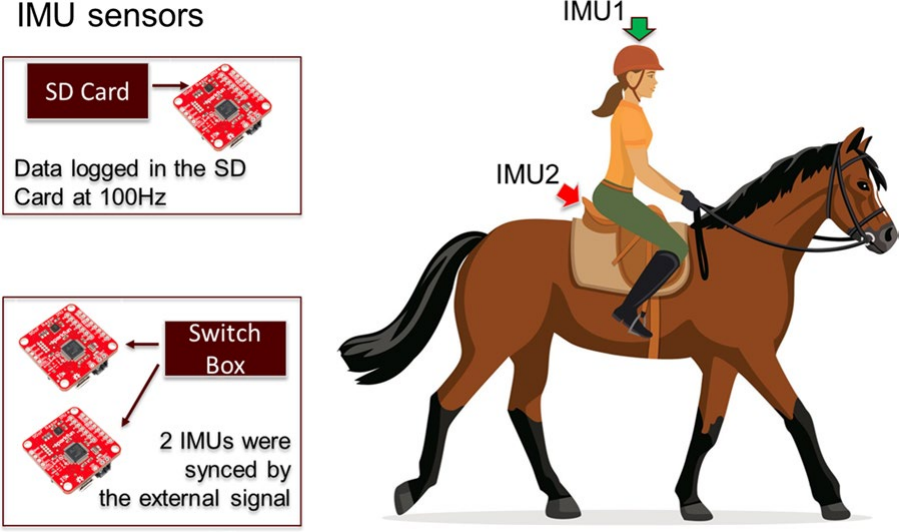
IMU sensors to capture the sinusoidal wave pattern of the horse’s gait at a walk [20] and to examine how the rider and the horse interact

The fourth participant ambulated with a rolling walker, had a decreased cadence, and found sit-to-stand transitions challenging, making the *TUG* impractical and necessitating a different assessment tool. The 10 Meter Walk Test (*10 mWT*) was chosen, which measures the time it takes a person to walk at a comfortable speed from markers at 2–8 m within the designated 10 m pathway. It is cost effective, easy-to-use, safe, and has been shown to have excellent inter-rater and intra-rater reliability [27].

#### Sensors

To examine how the riders and horses interact and to investigate the causes (i.e., kinetics) of movement (i.e., kinematics including displacement, velocity), one inertial measurement unit (IMU) (9DoF Razor, SparkFun, Boulder, Colorado, United States) was attached on the head/helmet of the rider. Another IMU was attached to the bareback pad at approximately lumbar vertebrae 4–5 junction for the two larger horses and at approximately lumbar vertebrae 5–6 junction for the two smaller horses (Fig. 2). The SparkFun 9DoF Razor was selected because it was tiny, lightweight and contained a board with a microprocessor, IMU and a microSD card. Since the Razor IMU was tiny and lightweight, it had minimal chance to distract the children with CP and the horse during the HPOT sessions. The IMU data on each Razor IMU were logged to the microSD card embedded to it with a sampling rate of 100 Hz. Before each HPOT session began, all Razor IMUs were synchronized by a single sync signal triggered by an external push button (Fig. 2).

**Fig. 3:**
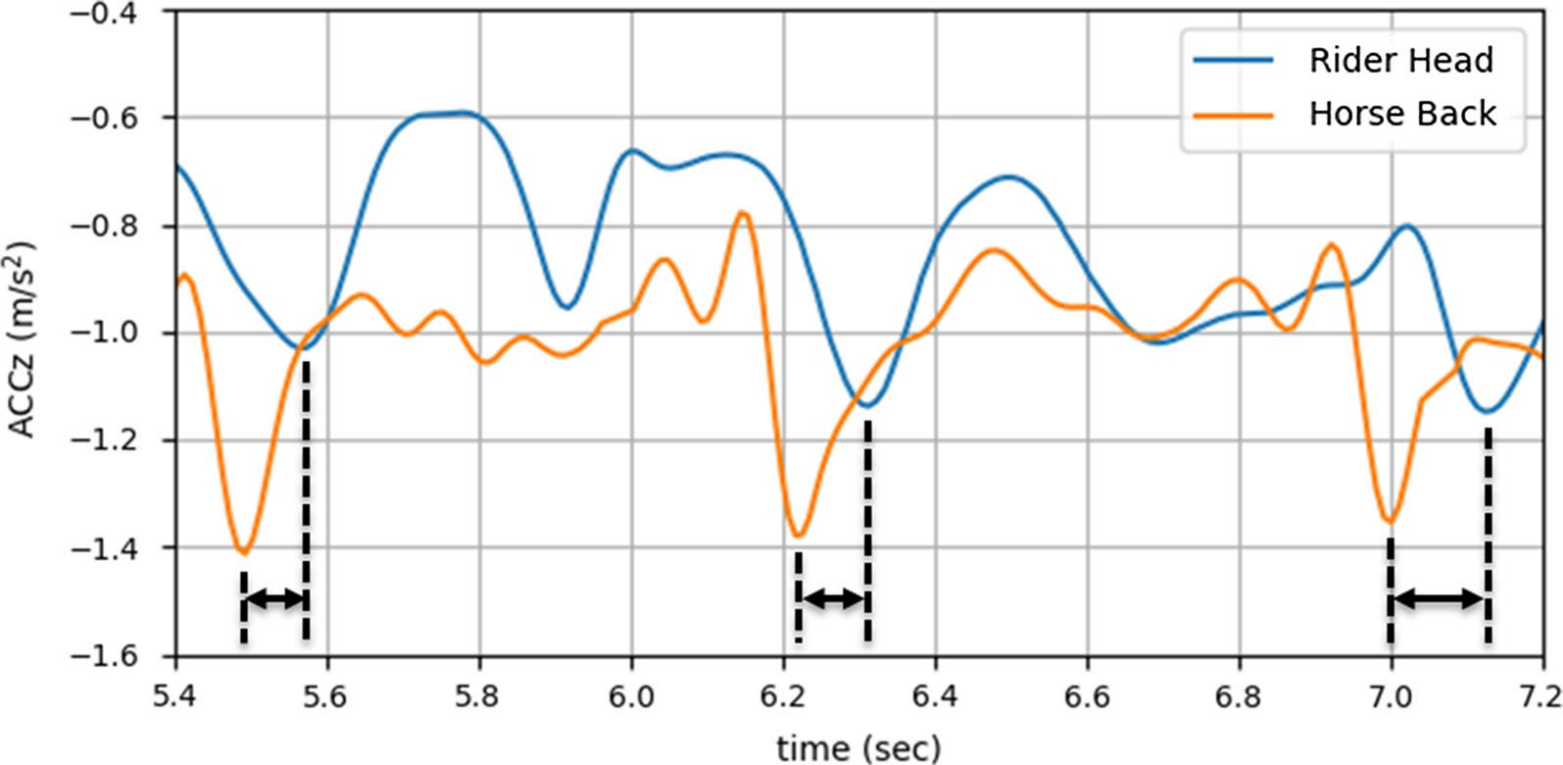
Sample plots of *ACCz* for both rider’s head (i.e., IMU1 from Fig. 2) and horse’s back (i.e., IMU2 from Fig. 2). *ACCz* from IMU1 (in blue) lags *ACCz* from IMU2 (in red)

**Fig. 4:**
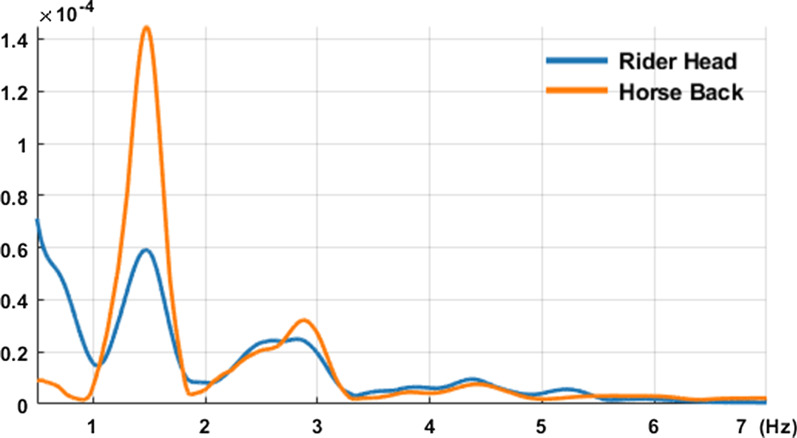
Power Spectral Density of ACCz from head and ACCz from horse back

**Fig. 5:**
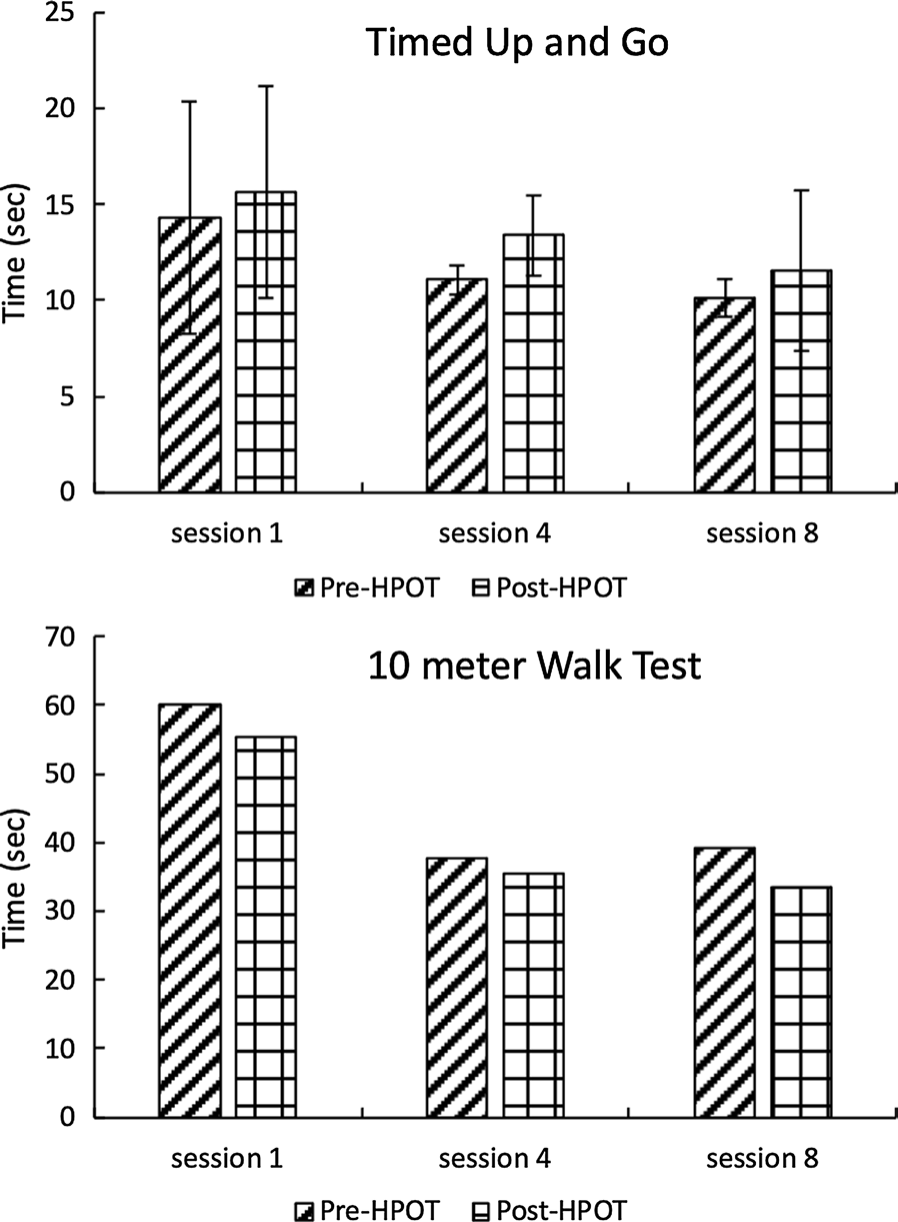
Bar graphs of the functional mobility tests. Top graph shows *TUG* results for participants 1–3 whereas bottom graph shows *10 mWT* for participant 4. Error bars in the top graph indicate one standard deviation. Bottom graph does not have the error bars since it involves with only one participant

#### Intervention during sessions

The horses were led by a trained horse handler and accompanied on each side by a physical therapist and an assistant. The equine partners were fitted with a saddle pad, bareback pad, girth, and side-pull or halter. Participants wore approved riding helmets and rode in a forward-astride position. The riding pattern was designed by the two physical therapists conducting the study, both Hippotherapy Clinical Specialist-certified by the American Hippotherapy Certification Board. The pattern was designed to maintain consistency of the movement patterns and was never altered. The trajectories of the horse and walking distances were controlled as much as possible between arenas.

**Fig. 6:**
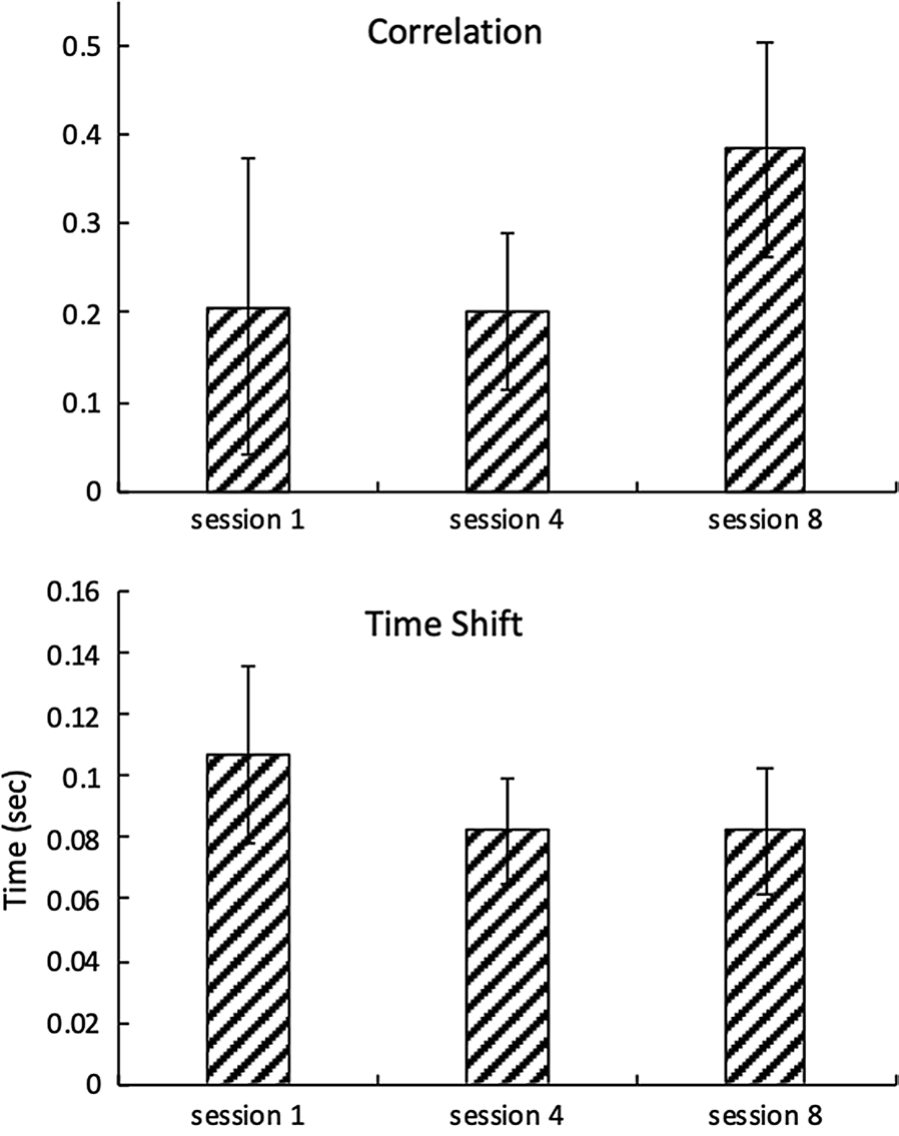
Maximum correlation (top) and time shift for the maximum correlation (bottom). Error bars indicate one standard deviation

**Fig. 7:**
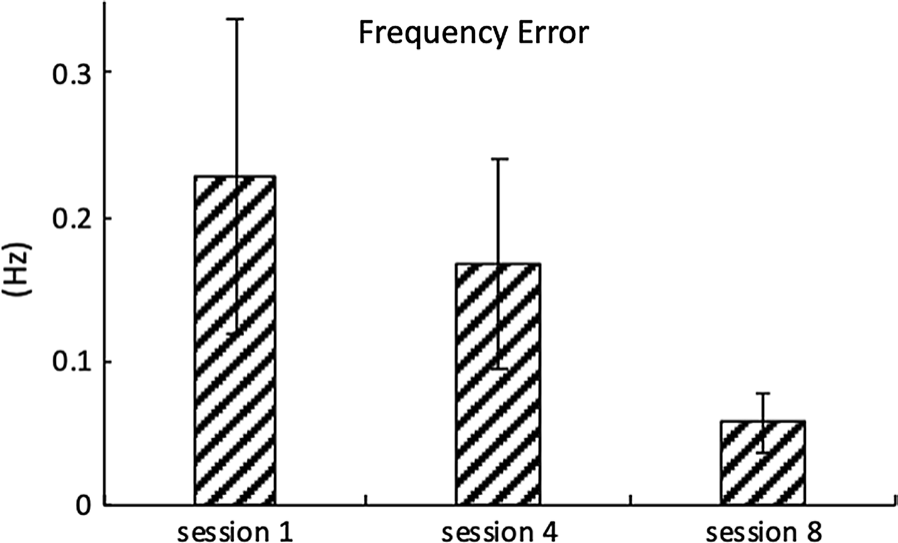
Root Mean Square Error (RMSE) between the peak harmonics of head *ACCz* and horse’s back *ACCz*. Error bars indicate one standard deviation

Eight 20-min physical therapy sessions incorporating HPOT were conducted (Fig. 1). A series of figure-of-eight patterns were made, at a steady pace, across the arena for the initial 10 min. For the second 10-min period, the horse continued the pattern, walking at the same steady pace but with walk-halt-walk transitions at 1-min intervals. Three of the four children were given a ring-shaped toy to hold with both hands during the second 10-min period, to reduce the impulse for upper extremity protective extension with changes in perturbations. The fourth child was not given a toy as she needed her hands on a weight-bearing surface to maintain stability. The first half of the session allowed the riders to feel to the slow, rhythmical, multi-dimensional aspect of the horse’s gait at a walk. The second part of the session further challenged the rider’s balance, righting reactions, and trunk control.

Throughout the session, the physical therapist monitored the participant’s position and midline orientation. If the rider shifted off midline, the physical therapist had the horse handler stop the horse so that the rider could regain midline orientation. Each rider needed a static surface to regain midline orientation, but with varying degrees of assistance.

### Data analysis

#### Variables related to functional mobility tests

The time (in seconds) taken to complete *TUG* were recorded. Alternatively, when the *TUG* was not feasible due to functional limitations of the participant, the time taken to complete *10mWT* were recorded. These functional mobility tests were measured once before and again after HPOT sessions on days 1, 4 and 8 for a total of six tests per participant (Fig. 1).

#### Variables related to interaction

To analyze how the riders and horses interact, we examined the vertical acceleration, *ACCz*, from all sensors for the following reasons. First of all, acceleration can be considered as an interaction force between the horse and rider normalized by the rider’s body mass. Several studies also have reported that leg acceleration and ground reaction force are highly correlated while running [30, 31]. Therefore, even though acceleration is a kinematic variable, it conveys the information on the cause of the movement, not like other kinematic variables including position, velocity, and orientation. Even though Uchiyama et al. [24] also investigated acceleration, they simply compared acceleration of human walking and acceleration of horse walking to examine the similarity of their walking, but didn’t study how horse’s walking affects human’s movement. Second, we decided to focus on the vertical direction since the vertical up-and-down movement of the center of mass (1) is dominant and energy-efficient [32, 33] and (2) involves with significantly larger impulse due to the gravity as opposed to any other directions [34]. *ACCz* indicates changes in gravity that generate physical changes in movements of the body [35], and may represent the interaction force normalized by the mass of the body. In this study, we analyzed the data from the first 10 min of the sessions (Fig. 1), when the equine movement was continuous, to observe the uninterrupted repetitive and rhythmical patterns. Data from the second half of the sessions will be analyzed in the future study.

For simplicity, we assumed that the signal from the horse’s back was the reference signal and that the signal from the rider’s head was affected by the reference signal. The cross-correlation between the reference *ACCz* and the *ACCz*’s from the rider’s head was studied. The correlation between the two signals indicated the similarity between the two, ranging from $-1$ to 1. Due to the nature of the interaction between the horse and the rider, the two signals exhibited a time difference (Fig. 3). The horse imparts movement to the rider and the rider’s body, as the recipient of that force, responds to the movement. Therefore, the time shift (in seconds) of the reference signal that produced the maximum correlation was also examined. The higher correlation and smaller time shift may indicate that two systems (i.e., the horse and the rider) synchronize temporally.

In addition, *ACCz* was analyzed in the frequency domain via the fast Fourier transform (FFT) to study the dominant frequencies of the signals. Specifically, harmonics, i.e., multiple peaks, of the transformed data were analyzed. Assuming that harmonics of the horse’s back were the reference signals, harmonics from the IMU on the rider’s head were compared (Fig. 4). The errors between the reference harmonics and the rider’s harmonics at these dominant frequencies were examined. Specifically, Root Mean Square Error (RMSE) was computed to study how much the rider’s harmonics were different from the reference harmonics [36, 37]. Smaller harmonics errors may indicate that two systems (i.e., the horse and the rider) synchronize spatially. No statistical analyses were performed due to small sample size ($n=4$) in this pilot study.

## Results

### Functional mobility tests

Participants 1–3 performed the *TUG* whereas participant 4 found sit-to-stand transitions challenging, making the *TUG* impractical. Therefore, participants 1–3 performed *TUG* and participant 4 performed *10 mWT*. On average, the times taken to finish the *TUG* decreased by 18.3% and 27.5% for session 4 and session 8 compared to session 1, respectively (Fig. 5). A few exceptions existed. For example, subjects 2 showed increased *TUG* after HPOT session 4 compared to session 1 whereas subject 3 showed increased *TUG* before HPOT session 4 compared to session 1.

Notably, the *TUG* results were more variable after the HPOT sessions (s.d.: 4.17) than before (s.d.: 3.56) (Fig. 5 top left vs. bottom left).Specifically, variability drastically reduced during sessions 4 and 8 for Pre-HPOT whereas variability remained relatively constant throughout the sessions for Post-HPOT.

The three participants who had hemiplegia ambulated without assistance but demonstrated diminished balance skills and decreased cadence. All wore bilateral ankle-foot orthotics (AFO). The youngest child had a submalleolar orthotic inside her AFO to increase ankle stability and walked with hip internal rotation on the right, her affected lower extremity. Following HPOT sessions, the internal rotation was less pronounced. The same held true for the pre-kindergarten child who demonstrated right hip internal rotation more before his HPOT sessions than when walking after his treatments. Anecdotally, the youngest child (age 32 months) did not comply with instructions to sit in the chair at the end of the test; instead, just prior to sitting she chose to go look for her mother.

Participant 4, who required a rolling walker and contact-guard assistance, demonstrated improved times on the *10 mWT* over the sessions (Fig. 5 right column). On average, the times taken to finish the *10mWT* decreased by 36.6% and 37.1% for session 4 and session 8 compared to session 1, respectively (Fig. 5). Further, at the end of her first HPOT session she appeared tired (i.e., increased drooling) and was easily distracted; at the conclusion of her eighth and final session, she was talkative and attentive. There was no variability measured for *10 mWT* since there was only one participant for it. This participant wore bilateral AFO’s. At the beginning of the study, she required maximum assistance with the rolling walker to prevent it from veering sharply to the right, and moderate-maximum assistance to prevent forward flexion at the trunk. By her last session, post-HPOT, she needed only minimum assistance to keep the walker on the straight-forward path to complete the test. Also, her trunk was more upright, demonstrating improved postural alignment and control. While not related to mobility, the child was very soft spoken as a result of scarring from ventilation tubes when she was an infant. The volume of her voice had consistently increased by the time she finished her HPOT session.

### Interaction: *ACCz*

Overall, the time series data from both IMU sensors tended to resemble each other as the HPOT session progressed. The maximum correlation between the reference signal (i.e., *ACCz* from horse’s back) and *ACCz* from rider’s head increased 84.7% for session 8 compared to session 1 (Fig. 6 left). Similarly, the time shift also decreased 23.3% and 23.3% for session 4 and session 8, respectively, compared to session 1 (Fig. 6 right).

Dominant frequencies were observed at around 1.5, 3.0, and 4.5 Hz for both the horses and the riders, which agrees with the literature [24] (Fig. 4). Components at the lower frequencies (e.g., less than 1 Hz) are the constant artifacts due to gravity, and thus are not considered for the analysis. The data revealed that as the physical therapy sessions utilizing HPOT treatments progressed, the dominant harmonics of *ACCz* for both the horses and the riders converged to each other, suggesting that all participants demonstrated an increase in synchronization with the horse during the horse’s movements at a walk. Of note, the Root Mean Square Error (RMSE) of the dominant peak frequencies of *ACCz* for both the horse’s back and the rider’s head decreased by 26.5% and 74.5% for session 4 and session 8 compared to session 1, respectively (Fig. 7). Interestingly, variability of the RMSE decreased by 32.1% and 81.1% for session 4 and session 8 compared to session 1, respectively (Fig. 7). Reduced RMSE mean and variability may indicate that the riders and the horses interacted in more consistent and synchronous ways.

## Discussion

Due to limited number of participants, statistical analyses could not be performed. Instead, mean and standard deviation (s.d.) were reported in the result section. In sum, with continued HPOT sessions, children with CP showed improved functional mobility (Fig. 5). For children with CP, functional deficits are often a result of poor postural control [6]. Yet motor skills improve when postural control improves [38]. HPOT may facilitate equilibrium and righting reactions through the variations in the horse’s velocity, direction, and stride length [14]. In a study by MacPhail et al. [13], the researchers noted that involuntary postural reactions of the trunk and head—specifically, equilibrium and righting reaction—were a result of the passive displacement of the rider’s center of gravity. The movement imparted to the rider when the horse is walking plays a crucial role in HPOT treatments.

With continued HPOT sessions, vertical movements (i.e., *ACCz*) of children with CP and horses appeared to become more synchronized (Figs. 6, 7). Participants may have become more familiar with the horse’s movement pattern. This observation is significant for therapists who may want to incorporate equine movement as a treatment strategy. One reason is that for children, motor learning requires the effective training of motor function [39]. Despite limitations, the child must problem-solve and be an active learner to obtain new age-appropriate skills [39]. Children differ from adults in that, typically, they are not trying to regain function as they lack a motor image of how to perform a new task [39]. To learn new motor skills, the new skill must be practiced multiples times, which may be why the horse’s gait at a walk can be an effective tool in gaining postural control. According to Janura et al. [40], a frequency of 90-100 impulses per minute are imparted to the rider, providing many opportunities for postural adjustments, even within a limited time period. This is significant since proximal stability and postural control are the foundation on which children learn functional motor skills [19].

Postural control is affected by sensory information [41]. Children with CP often have impairments in sensory processing [41]. During HPOT the participant is experiencing multiple impulses per minute and reacting to such movements [17]. This offers cognitive, limbic, and physical stimulation [10, 42], as well as visual, vestibular, and the somatosensory system [17]. Combined, these concentrated stimuli to the participant may facilitate development of new movement strategies in a way not offered in a more traditional PT session [10].

Another factor supporting HPOT as a treatment strategy is that the movement of the horse at a walk follows a sinusoidal wave pattern [20, 38]. This pattern puts a demand on the rider’s automatic postural responses as they must coordinate and control their movements [13, 19]. Also, the dynamic treatment and changing environment may affect multiple systems, including vestibular and proprioceptive systems [12, 14]. With the dynamic movement on the horse, compensatory postural strategies may be reinforced or explored [17, 19]. The cyclical and repetitive movements provide numerous opportunities for practice of postural adjustments [12]. Silkwood-Sherer et al. [17] suggested that with this type of therapy children can improve reactive and anticipatory postural control strategies in response to complex sensory input. Maintaining postural control while simultaneously moving through space and adjusting perceptual skills, facilitates the refinement and exploration of new movement patterns, which in turn, enhances functional mobility [17].

A third factor in favor of integrating HPOT into physcial therapy treatments is that the horse’s movement at a walk simulates the human gait pattern [16, 20, 24, 38]. Many children with CP have diminished ambulation skills, due in part to poor balance control [7, 38]. Liao et al. [38] found that rhythmic weight-shift training may facilitate improved walking performance for children with CP. It appears that HPOT may provide an opportunity for balance skills and ambulation skills to be addressed simultaneously for this population.

Last, many children with CP are restricted by slow gait speed which is one measure of walking performance [1, 38, 39]. Quality of life and functional ability are also linked to walking [5]. While the findings from this study are not statistically significant, it is noteworthy that the participant who performed the *10mWT* demonstrated a considerable improvement in gait speed. Her walking speed improved substantially during the course of the study and her parents reported a significant increase in her transfer skills at home. These results corroborate the findings observed by Casady and Nichols Larson [12] that HPOT may influence skill acquisition of motor tasks in daily functional tasks.

To our knowledge, this is the first study to investigate the interactive forces produced by the movement patterns of a horse at walk with a rider, a child with CP. While the findings are encouraging, this study had several limitations: (a) small sample size; (b) range in ages and ability levels of participants; (c) two functional mobility tests were administered; (d) only one of the three dimensions of the horse’s movement pattern at a walk was analyzed; (e) causal relation between enhancements in functional mobility and synchronized interaction may not be determined; and (f) the observed synchronized interaction may not tell us whether horses affected the children with CP more or vice versa. Future studies will examine these factors to extrapolate the findings to a broader population of children with CP. Also, future research could focus on other planes of movement imparted to the rider by a horse at walk to better understand the dynamics of the interaction of the forces during a HPOT session. In addition, technically, more sophisticated alignment procedures for the IMU sensors and the corresponding preprocessing will be performed to ensure easier data processing procedure and more enhanced data quality.

## Conclusion

Benda et al. [10] noted that in addition to developing skills, HPOT provides social, emotional, cognitive, and physical stimulation in a way not typically seen in conventional treatment. HPOT has been shown to positively influence skill acquisition, including balance and postural control, the foundations of movement. In this study, we questioned whether HPOT can lead to improved functional mobility in children with CP. Outcome measures demonstrated a trend towards improvements in the functional mobility of participants, indicating a positive response to the physical therapy treatments incorporating equine movement.

The findings from this study suggest that with continued HPOT sessions, participants appeared to become more familiar with the horse’s movement. The horse’s gait at a walk is consistent, cyclical, rhythmical, bilateral, and symmetrical. Given that it also mimics the human gait [20, 24, 38], the increasing synchronization between horse and rider suggests that HPOT is a viable physical therapy treatment tool to facilitate functional mobility goals. Despite the limited number of participants, this study may provide a useful baseline for future work.

## Data Availability

Summary data of the study are included on GitHub repository [43]. All data collected in the study are available from the corresponding author upon reasonable request.
